# Reactive Task Performance Under Varying Loads in Division I Collegiate Soccer Athletes

**DOI:** 10.3389/fspor.2021.707910

**Published:** 2021-10-13

**Authors:** Lauren E. Rentz, Cheryl L. Brandmeir, Bobby G. Rawls, Scott M. Galster

**Affiliations:** ^1^Rockefeller Neuroscience Institute, West Virginia University, Morgantown, WV, United States; ^2^Department of Human Performance, Division of Physical Therapy, West Virginia University, Morgantown, WV, United States

**Keywords:** choice reaction time, simple reaction time, soccer, performance, load-bearing, training demands, athletes

## Abstract

This study was conducted to identify whether team-wide or positional differences exist in simple or choice reactivity of collegiate soccer athletes when completed under various loads. Much research exists surrounding the assessment of reaction time in the general population, but given variations in training, little insight exists surrounding how unique and elite populations may differ based upon performance demands and task translatability to training. Reactive performance was assessed using the Dynavision D2 in 24 female soccer players (19.73 ± 1.05 years old) from a team within a power five conference of the National Collegiate Athletic Association. Evaluated loads included two conditions of simple reactivity (no additional load and with a concurrent lower body motor task) and three conditions of choice reactivity (no additional load, with a concurrent lower body motor task, and prolonged durations). Paired *t*-tests and ANOVAs were used to identify differences in task performance based upon load and positional group. No significant load-based or positional differences existed in measured simple reaction times. Performances in choice reaction tasks across the team were found to be slower when completed across extended durations (*p* < 0.0001) and faster when completed concurrent with an added balance task (*p* = 0.0108), as compared to performance under normal conditions. By assessment of positional differences, goalkeepers tended to be slower than other positions in reactivity during choice tasks, despite no differences existing in simple task performance. Given the unique population utilized herein, measured reactivity in different tasks suggests a strong relation to the training demands of soccer, as well as those of goalkeepers as compared to field positions. Findings suggest that sport and positional demands may be substantial contributors to population- and individual-based reactivity performance.

## Introduction

Defined as the length of time it takes for a person or system to respond to a stimulus or event, reaction time is a regularly overlooked aspect of performance as compared to the efficacy of conditioning and sport-specific skills. Quick reaction time is often a key contributor to achieving optimal sports performance and reducing the risk of injuries of varying types (Honda et al., 2018; Clark et al., 2020). Key physical attributes of collegiate soccer players include functional characteristics such as speed, power, and agility; however, team-based ball sports, such as soccer, involve a dynamic environment and rely on training anticipatory skills to improve reaction times. Specifically, soccer players train to anticipate the speed and direction of the ball. Beyond performance, reaction times can also contribute to sports safety and, thus, sports longevity; advanced neurocognitive reactivity has been found to decrease the risk of musculoskeletal injury and concussive events, both of which are of significant concern for soccer players (Wilkerson, 2012;Herman et al., 2015).

Athletes spend a large portion of their time training to reach peak performance. Drills to improve physical and mental performance are often incorporated, including those of strength, balance, core activation, dual tasking, and automaticity (Hoff and Helgerud, 2004; Williams and Ericsson, 2005; Jajtner et al., 2013; Williams et al., 2020). While some tasks are solely sport, and even position-specific, others are more holistic with the global focus of improving efficiency through faster neuromuscular pathways in the trained athlete (Hoff and Helgerud, 2004; Appelbaum and Erickson, 2018).

Numerous studies have been conducted to assess and potentially explain situational differences in reaction time. Assessments of reactivity have been studied separately for reaction speeds of visual and auditory stimuli in athletes, with faster reaction times resulting from differing stimuli for athletes of various sports (Breen et al., 1969; Hascelik et al., 1989; Baur et al., 2006; Nuri et al., 2013). In soccer players specifically, the tendency for faster visual reactions has been attributed to their dependence on visual cues for specific gameplay (Spierer et al., 2011). In studies that have compared the different roles on the team, goalkeepers have been found to have faster visual reaction times than other positions (Ruschel et al., 2011). It has been postulated that this tendency for goalkeepers to have faster reaction times than defenders, midfielders, and forwards is due to specialized training dedicated to reactive and short burst agility (Taskin et al., 2016).

Beyond general reactivity, it is important to consider the situational differences that may affect the way in which the brain responds to stimuli. Simple reactivity, in which a known response occurs in a predetermined way, primarily involves the neuromuscular activation to physically produce a response. In contrast, choice reactivity concerns differential responses based upon unknown conditions. The addition of spatial positioning variance and perceptual demands during choice reactivity, which is lacking in simple reactivity, results in activation of different brain regions; in addition to increased activation, bilateral hemispheric reliance becomes a greater factor during choice reactivity (Anzola et al., 1977; Heilman and Van Den Abeli, 1979). Resultingly, simple reaction times tend to be much faster as compared to choice, with performance slowing as complexity increases (Pins and Bonnet, 1996). Thus, variations in the conditions for reactivity that are demanded by differing sports or between positions within a team may contribute to individual and population diversity in reactive performance (Serrien et al., 2006). For soccer athletes, reactivity is demanded under different conditions than that of many other sports. Unlike the simple reaction occurring at the start of a snap in American football or the sound of the starter pistol for track athletes, the environment during a game of soccer is dynamic; the speed and spacing in the field of play are always changing, requiring athletes to be intimately aware of their surroundings at all times and reacting mostly to actions with decisions and both temporal and spatial elements. Further, these reactions by soccer players are typically occurring while the athlete traverses the field rather than from an isometric position, combining the need for dynamic cognition with concurrent lower body motor function.

The present study aims to assess simple and choice reaction times in a National Collegiate Athletic Association Division I Women's Soccer team. While reaction times have been thoroughly investigated in the general population, the sport-specific demands of this unique sample present questions surrounding the transferability of those findings to other populations and athletes of other sports. Further, few studies have assessed these aspects of performance across the various soccer positions to determine if differences exist. Considering the high relation of reactive performance to gameplay, simple and choice task performance during extended durations and with concurrent lower body motor activity are explored in a unique sample; it is hypothesized that performance in this population will vary from that of previous research based upon variations in performance and training demands of the evaluated sample.

## Materials and Methods

### Participants

Twenty-five Division I collegiate Women's Soccer athletes participated in the testing, which included all current members of the team; one athlete was excluded due to previous participation in visual training. The resulting 24 participants (19.73 ± 1.05 years old) had never participated in any type of visual training or had tested with the Dynavision D2 technology; of these participants, three were goalkeepers, seven were defenders, seven were forwards, and seven were midfielders. None of the participants had a current musculoskeletal injury, nor were they actively concussed at the time of testing. Prior to participation, all participants provided their written informed consent to participate. The study was approved by the West Virginia University Institutional Review Board (protocol #2009105273) and all study procedures were conducted in accordance with the Declaration of Helsinki Guidelines.

### Data Collection

**Figure 1:**
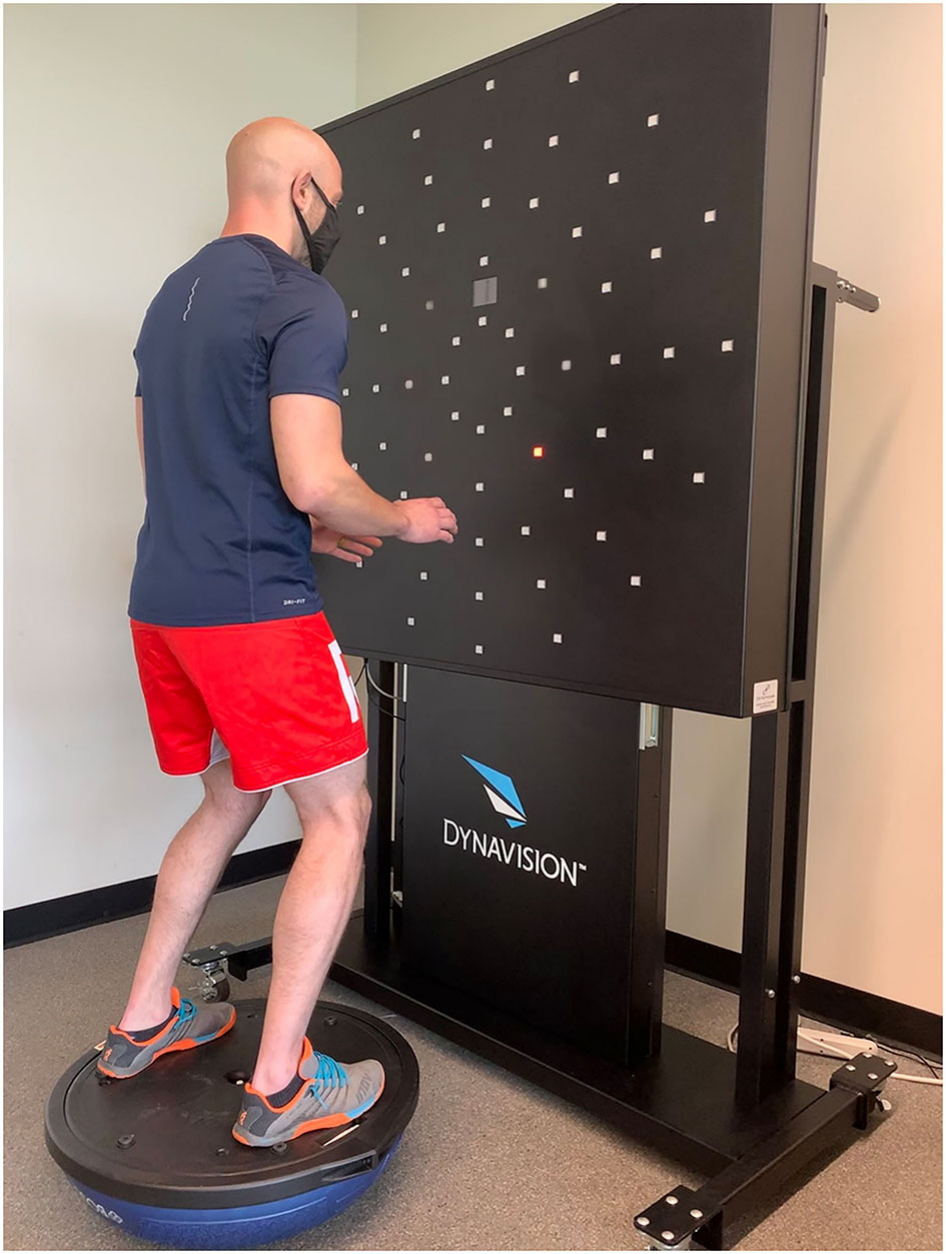
Physical conditions during balance tasks. Participants were positioned in front of the Dynavision D2 board atop a Bosu Ball for two of the five tasks.

Procedures were explained to all participants, and each were given the opportunity to familiarize themselves with the Dynavision D2 (“Dynavision”) board (Dynavision Global Holdings LLC, West Chester, OH, USA). The Dynavision D2, seen in Figure 1, has 64 buttons arranged into five circular rings, spanning outward from the center of the board; previous research has demonstrated its utility in measuring participant response patterns to various types of stimuli as an assessment tool for vision, cognition, reaction time, and motor function (Esposito et al., 2008). While standing ~16 inches away, the board was adjusted to the height of the individuals to ensure consistent placement across all participants.

The participants completed a protocol consisting of five assessments using the Dynavision board, lasting ~25 min in duration. All testing was completed in a quiet area with consistent lighting. During testing, participants were asked to wear contacts rather than glasses, if vision correction was required, to avoid frames from contributing to peripheral blind spots in the visual field, as well as attempt to reduce variation in vision accuracy. The testing protocol consisted of the following tasks, which utilize preprogrammed Dynavision modes that have been widely used in the literature, each separated by a 30-s break (Klavora et al., 1995; Jajtner et al., 2013; Bigsby et al., 2014; Feldhacker and Molitor, 2019; Feldhacker et al., 2019; Stone et al., 2019; Blackwell et al., 2020; Hunzinger et al., 2020). Each of the tasks and the conditions under which they were completed are defined below:

1. Four-minute proactive task (also known as ^*^Proactive Endurance, 4 min)
2. One-minute proactive task (also known as ^*^A test or ^*^Proactive, 1 min or Mode A)
3. Reaction Test (also known as ^*^Reaction Test [RT] or Mode D)
4. One-minute proactive task (also known as ^*^A test or ^*^Proactive, 1 min or Mode A) while balancing on a Bosu Ball
5. Reaction Test (also known as ^*^Reaction Test [RT] or Mode D) while balancing on a Bosu Ball

The first three tasks were completed while standing on the ground in a stance approximately shoulder-width. After the first three tasks, participants stepped onto the flat, platform side of a Bosu Ball (BOSU, Ashland, OH, USA) and stood with their feet at shoulder-width apart to encourage lower extremity neuromuscular activation, as seen in Figure 1. Once atop the ball, the height of the Dynavision board was raised to account for the added height of the Bosu Ball. Participants were allowed ~30 s to obtain their balance and familiarize themselves with the Bosu Ball prior to completing the final two tasks of the protocol. This added balance requirement of standing on the Bosu Ball augmented activation of the lower body and postural muscles, which more closely resembles sport-specific conditions of the population where they concurrently undergo lower body motor functions and has previously been utilized in similar methodology (Bigsby et al., 2014).

#### Simple Reactivity

The Reaction Tests (RTs) measured simple reaction performance, and each consisted of three tasks that were completed with six repetitions each from the left and right hand. At the start of each task, participants were instructed to hold down a reference button in the center of the board until they saw a new light appear from a predetermined region of the board. Once they saw the new light, participants were to remove their hands from the reference button and dismiss the light as quickly as possible. All participants completed the test trials in the same, predetermined order as programed by the Dynavision software. Reaction times were recorded for the visual reaction time, which was measured from the time the light illuminated to the initiation of the motor movement, and for the motor reaction time, which was measured from the initiation of the motor activity to the successful dismissal of the light. The physical reaction time was also calculated, which is the sum of the visual and motor reaction times and represents the total time for task completion. Six repetitions of each task were completed using each hand and the averages for each were recorded. Performance in all tasks and both hands were averaged for inter and intra-individual comparison. The first RT acted as the *Single-load* condition, and the second RT acted as the *Dual-load* condition.

#### Choice Reactivity

Proactive tasks measured choice reaction performance and involved lights that illuminated one at a time at random on the board, of which participants were asked to locate and press as quickly as possible. A new light appeared on the board at a new random location immediately following the dismissal of a light. Participants were instructed to hit as many lights as possible throughout the duration of the task, and to act as quickly as possible. The 4-min proactive task acted as the *Endurance* condition due to the extended task duration as compared to the 1-min proactive task. Similarly, the two 1-min proactive tasks were completed under different loads: *The single-load* condition and the *Dual-load* condition. The *Single-load* condition was completed while standing under normal conditions, whereas the final 1-min proactive task acted as the *Dual-load* condition, in which the cognitive task was combined with a physical load of standing atop the Bosu Ball.

### Statistical Analysis

Data from the tests were exported from the Dynavision unit for each participant and were compiled along with positional information. Data analysis involved the use of Microsoft Excel version 16.44 (Microsoft Corporation, Redmond, WA, USA) and JMP Pro version 14.0.0 (SAS Institute Inc., Cary, NC, USA), where alpha levels were set *a priori* at 0.05. Paired *t*-tests were conducted to identify differences in the proactive or RT task conditions. All positions were confirmed to have variables with a normal distribution as assessed through the Shapiro-Wilk goodness of fit test. Different task conditions, time comparisons, and positional groups were checked for unequal variances using the Brown-Forsythe test and were determined to have equal variance. Mixed model ANOVAs were employed to test differences between position (between variable) and task performance (within variable); each model involved evaluation for the main effect of each variable and interaction between the two variables. Multiple comparisons were made using Tukey honestly significant difference test (HSD) for detection of any significant pairwise differences within the analyses, of which adjusted degrees of freedom and *p*-values are reported.

## Results

### Task Results

In regards to performance during the different proactive test conditions, a paired *t*-test for the entire team indicated a significant difference [*t*_(23)_ = −6.289, *p* < 0.0001] in the average reaction time of 0.81 ± 0.06 s during all hits in the *Endurance* task as compared to the 0.77 ± 0.06 s average during the *Single-load* proactive task. There was also a significant difference in total reaction time for the two 1-min proactive task conditions [*t*_(23)_ = −2.775, *p* = 0.0108] as indicated by a paired *t*-test; the team, on an average, was faster during the *Dual-load* condition averaging 0.74 ± 0.06 s per hit as compared to the 0.77 ± 0.06 s average during the *Single-load* condition. Despite this, the number of hits was not significantly different between the *Single-load* (78.75 ± 5.33 hits) and the *Dual-load* (80.75 ± 7.04 hits) conditions [*t*_(23)_ = 1.716, *p* = 0.0996].

During the proactive *Endurance* task, the number of hits recorded across the team during each of the 4 min was found to have a significant main effect of time [*F*_(3, 25.2)_ = 5.044, *p* = 0.0071]; the team averaged 72.08 ± 5.41 hits during the first minute, 74.54 ± 5.49 hits during the second minute, 73.88 ± 7.68 hits during the third minute, and 74.67 ± 6.30 hits during the fourth and final minute of the task. Pairwise comparisons indicated significantly more hits to be recorded during the fourth minute as compared to the first minute [*t*_(25.2)_ = −3.53, *p* = 0.0083].

**Figure 2:**
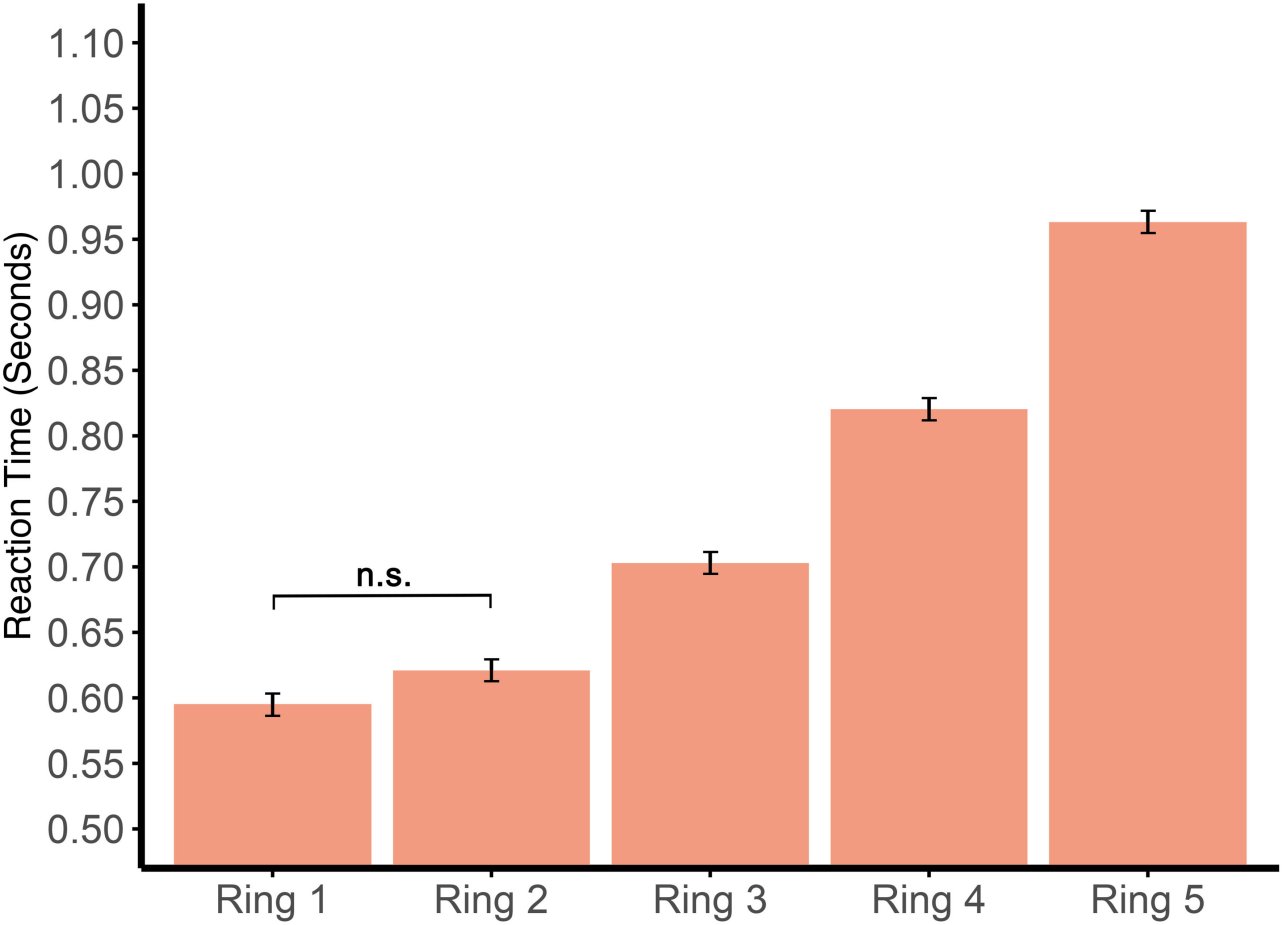
Average reaction times by location. The figure demonstrates the average reaction times for all participants in the five rings of the Dynavision board across all three proactive tasks. Ring 1 is the innermost ring of the board, with each ring progressing outward toward the outermost ring, Ring 5. All pairwise comparisons indicated significant differences except between Ring 1 and Ring 2, which is denoted by *n.s*.

During all three proactive tasks, reaction times were considerably slower for stimuli the further they moved outward from the center, which is demonstrated in Figure 2. Across the three tasks, all of the rings were significantly different from each other [Ring 2 vs. Ring 3, *t*_(351.9)_ = −6.55, *p* < 0.0001; Ring 3 vs. Ring 4, *t*_(351.9)_ = −9.38, *p* < 0.0001; Ring 4 vs. Ring 5, *t*_(351.9)_ = −11.42, *p* < 0.0001], except for Rings 1 and 2, the two innermost rings, which were not significantly different [*t*_(351.9)_ = −2.08, *p* = 0.2328]. Average reaction times across the three tasks were 0.60 ± 0.04 s for Ring 1, 0.62 ± 0.05 s for Ring 2, 0.70 ± 0.04 s for Ring 3, 0.82 ± 0.07 s for Ring 4, and 0.96 ± 0.09 s for Ring 5.

During the two RTs, there were no significant differences in visual, motor, or combined physical reaction times between the *Single-load* and *Dual-load* conditions. As a team, mean visual reaction times were 0.38 ± 0.04 s, motor reaction times were 0.27 ± 0.03 s, and physical reaction times were 0.65 ± 0.06 s during the *Single-load* condition. During the *Dual-load* condition, the team had a mean of 0.38 ± 0.04 s for visual reaction time, 0.27 ± 0.03 s for motor reaction time, and 0.65 ± 0.06 s for physical reaction time.

### Positional Results

**Figure 3:**
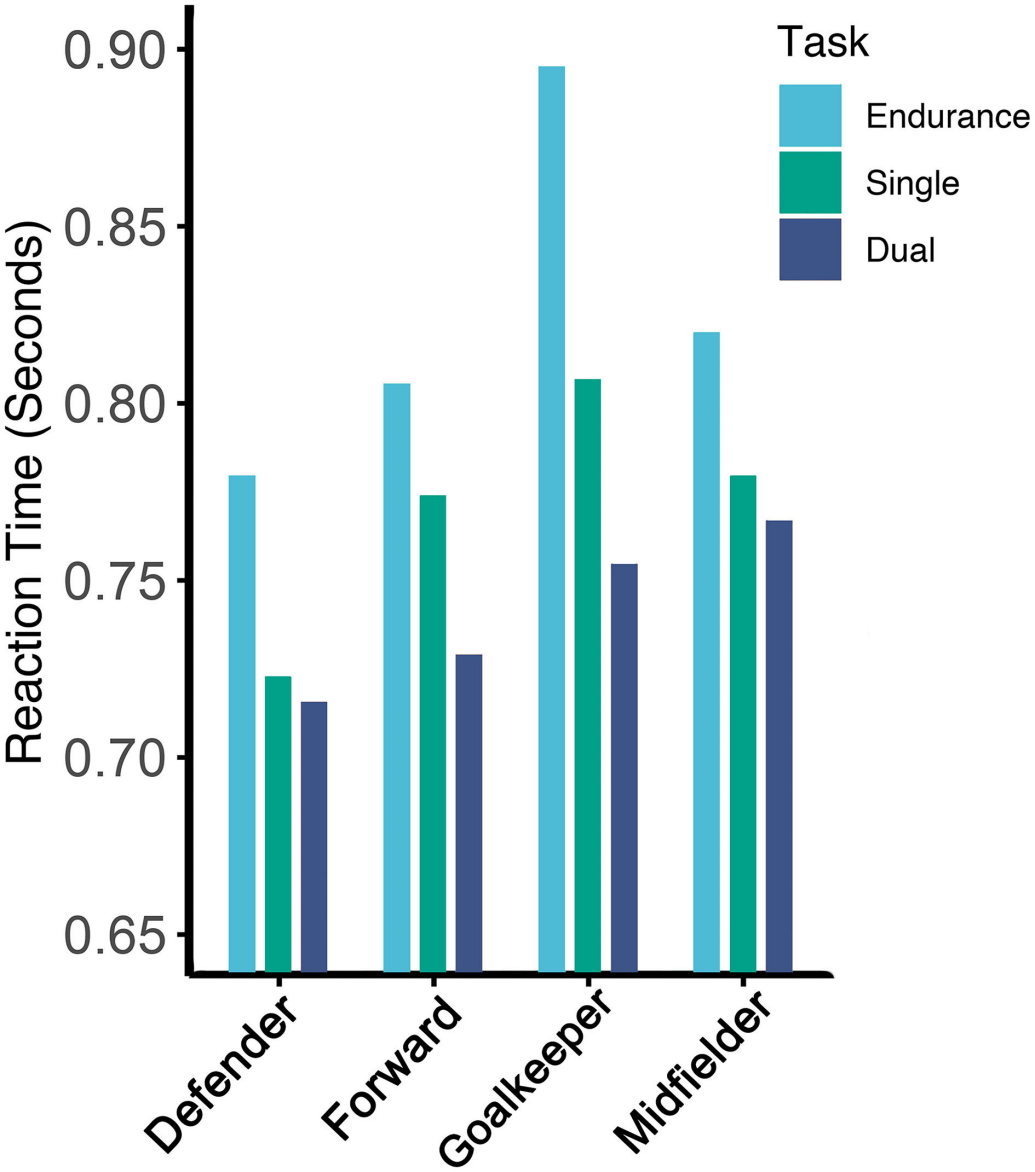
Total proactive reaction time by position. It demonstrates positional means in total reaction time, in seconds, by position during each of the three proactive tasks.

During the three proactive tasks, a two-way mixed model ANOVA indicated a significant main effect of task [*F*_(2,40)_ = 50.386, *p* < 0.0001] and a significant interaction between position and task [*F*_(6,40)_ = 2.456, *p* = 0.0408]; this interaction can be seen in Figure 3, and mean total reaction times for each position during the three proactive tasks are listed in Table 1. In goalkeepers, significant pairwise differences existed between reaction times during the *Endurance* task to both the *Single-load* [*t*_(40)_ = 3.95, *p* = 0.0142] and *Dual-load* tasks [*t*_(40)_ = −6.29, *p* < 0.0001], but no differences between the *Single-load* and *Dual-load* tasks. Defenders demonstrated the same pairwise differences between the *Endurance* task to both *Single-load* [*t*_(40)_ = 3.88, *p* = 0.0170] and *Dual-load* [*t*_(40)_ = −4.37, *p* = 0.0043] tasks, despite no differences in *Single-load* and *Dual-load* tasks. Midfielders and forwards only demonstrated significant pairwise differences between the *Endurance* and *Dual-load* conditions [midfielders, *t*_(40)_ = −3.64, *p* = 0.0324; forwards, *t*_(40)_ = −5.23, *p* = 0.0003].

**Table 1:** Total average reaction time during proactive tasks.

**Position**	**Endurance**	**Single**	**Dual**
Defenders	0.779 ± 0.040	0.723 ± 0.036	0.715 ± 0.063
Forwards	0.805 ± 0.053	0.774 ± 0.054	0.729 ± 0.051
Midfielders	0.820 ± 0.051	0.779 ± 0.061	0.767 ± 0.070
Goalkeepers	0.895 ± 0.084	0.807 ± 0.086	0.754 ± 0.079
Team	0.813 ± 0.061	0.765 ± 0.060	0.739 ± 0.063

*Mean ± SDs of average reaction times in seconds for every hit recorded during each of the three proactive tasks*.

**Figure 4:**
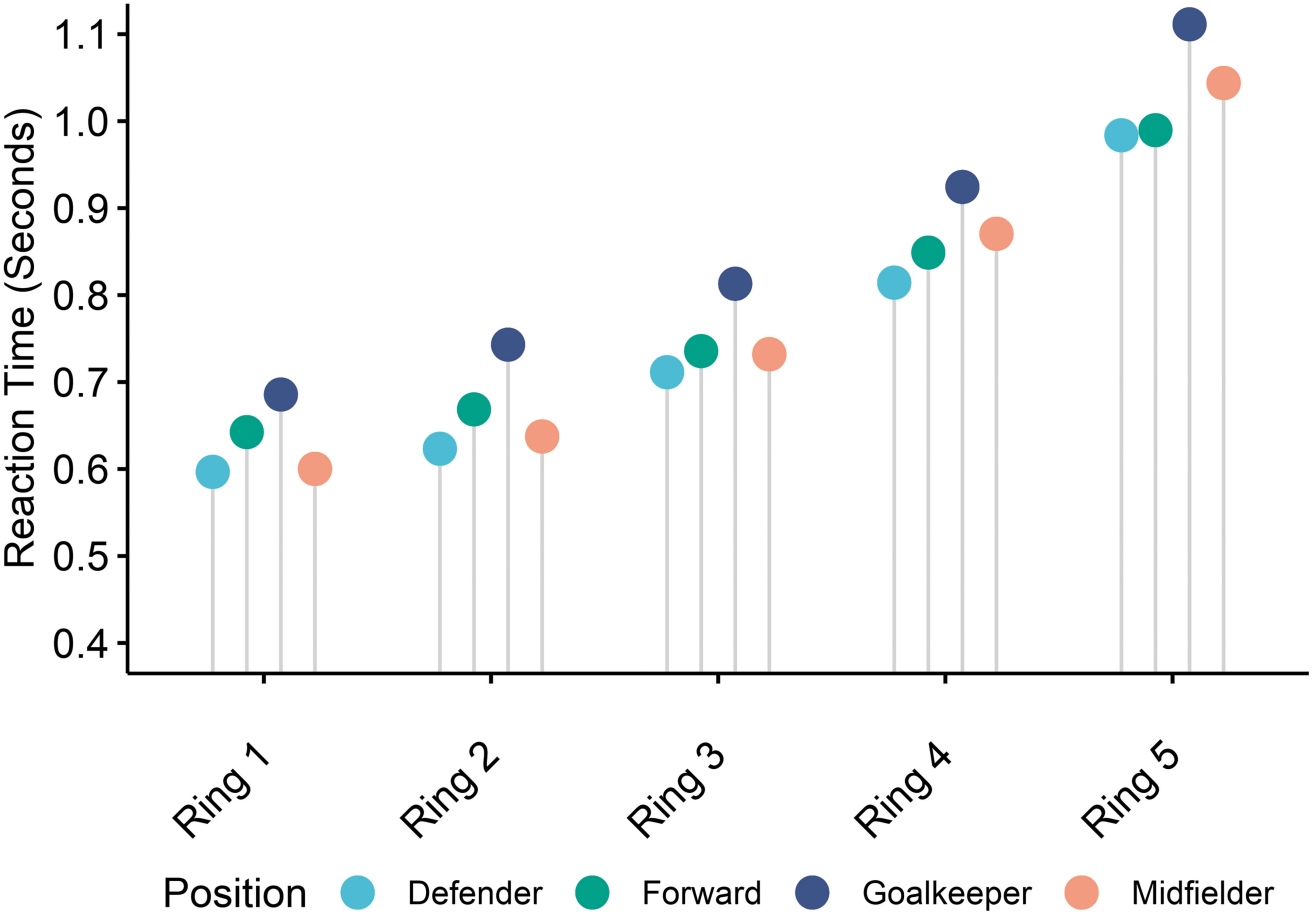
Average reaction time by region during the *Endurance* task. The figure demonstrates the average reaction time in seconds for each of the five rings of the Dynavision board across positions during the *Endurance* task.

During the *Endurance* task, a two-way mixed model ANOVA indicated significant differences in reaction time between positions in the different rings of the Dynavision board, which can be seen in Figure 4. There were significant main effects of position [*F*_(3,20)_ = 3.714, *p* = 0.0284] and ring [*F*_(4,80)_ = 261.661, *p* < 0.0001] on average reaction times, but not a significant interaction between position and ring. Average reaction times relative to all five rings were significantly higher in goalkeepers as compared to defenders [*t*_(20)_ = −3.34, *p* = 0.0161], but not midfielders [*t*_(20)_ = 2.40, *p* = 0.1103] or forwards [*t*_(20)_ = −2.39, *p* = 0.1118], as determined through pairwise comparisons. Two-way ANOVA's comparing reaction time by position and ring also indicated significant main effects of the ring during the *Single-load* [*F*_(4,80)_ = 168.302, *p* < 0.0001] and *Dual-load* [*F*_(4,80)_ = 125.885, *p* < 0.0001] proactive tasks, however, no significant main effects of position or interactions between position and ring.

**Figure 5:**
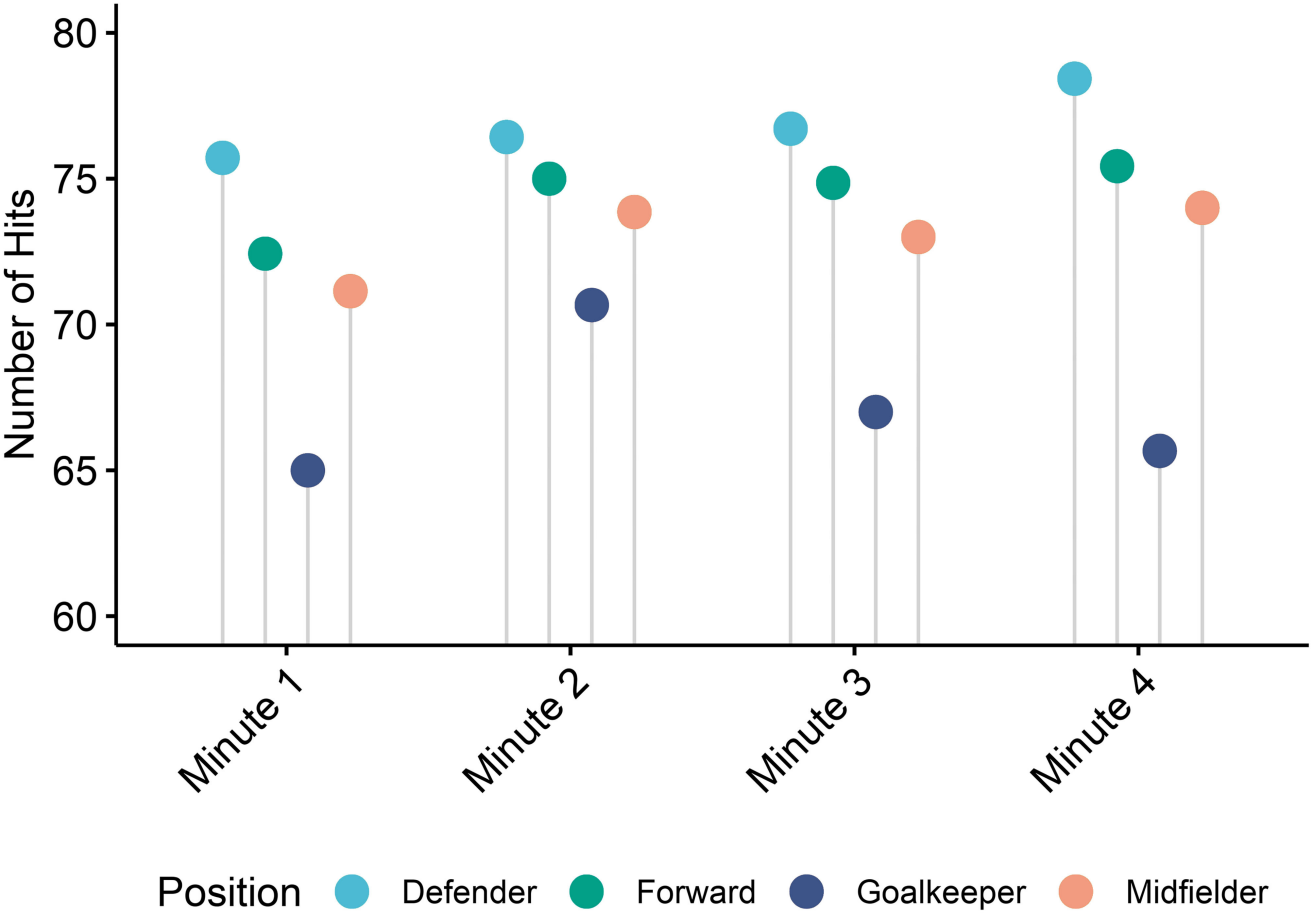
Hits during each minute of the *Endurance* task. The figure demonstrates the mean number of hits recorded for each position during each of the 4 min of the *Endurance* task.

When assessed by time, there were significant differences between the positions in the number of hits recorded per minute throughout the *Endurance* task as indicated by a two-way ANOVA, which is depicted in Figure 5. There was a significant main effect of position [*F*_(3,21.7)_ = 3.158, *p* = 0.0453] and time [*F*_(3,27.5)_ = 4.155, *p* = 0.0150] during the task. Goalkeepers, on average, responded with the lowest number of hits during each of the 4 min of the task; while the other positions maintained a consistent speed throughout the 4 min, the goalkeepers tended to peak during the second minute and responded with fewer hits as the task progressed. Goalkeepers responded with significantly fewer hits throughout all time points within the task as compared to defenders [*t*_(21.7)_ = 3.08, *p* = 0.0298] as indicated through pairwise comparisons. In total, goalkeepers averaged 268.3 ± 26.9 hits across the 4 min, midfielders averaged 292.0 ± 19.1 hits, forwards averaged 297.7 ± 20.4 hits, and defenders averaged 306.0 ± 15.9 hits.

During the two RTs, there were no significant differences in visual, motor, or physical reaction times across positions. None of the positions had significantly different reaction times of any type between the *Single-load* and *Dual-load* conditions. Average reaction times for each component of both conditions are listed in Table 2.

**Table 2:** Performance during the Reaction Tests (RTs).

**Condition**	**Position**	**Visual**	**Motor**	**Physical**
Single Task	Defenders	0.382 ± 0.037	0.249 ± 0.029	0.630 ± 0.051
	Forwards	0.385 ± 0.039	0.274 ± 0.047	0.658 ± 0.085
	Midfielders	0.381 ± 0.040	0.281 ± 0.017	0.661 ± 0.050
	Goalkeepers	0.367 ± 0.027	0.253 ± 0.027	0.619 ± 0.040
Dual Task	Defenders	0.382 ± 0.051	0.269 ± 0.031	0.650 ± 0.059
	Forwards	0.389 ± 0.046	0.276 ± 0.037	0.665 ± 0.062
	Midfielders	0.371 ± 0.034	0.279 ± 0.027	0.651 ± 0.060
	Goalkeepers	0.392 ± 0.017	0.251 ± 0.014	0.642 ± 0.031

*Average visual, motor, and physical (summation of visual and motor) reaction times for RT tasks under both conditions are listed in seconds for each position in terms of Mean ± SD*.

## Discussion

The purpose of this study was to assess performance during various task-loading conditions in a unique athletic population. In addition to identifying if differences exist between team positions, this study aimed to examine whether population-based differences exist, with consideration to other literature. With the crucial reliance on reactivity and extended bouts of attention in soccer players and many other athletes, it is of high priority that the effects of training relative to situational demands are well-understood.

Relative to other studies, performance in the *Single-load* ^*^A task and the *Single-load* RT were comparable to previous findings, indicating normative values for this population during their first attempt at this protocol (Clark et al., 2015; Feldhacker and Molitor, 2019; Feldhacker et al., 2019; Blackwell et al., 2020; Hunzinger et al., 2020). Most notably, performance in the *Single-load* ^*^A test was found to be nearly the same as those previously found in collegiate soccer athletes for women, as well as visual and motor RT speeds determined from a different study (Jajtner et al., 2013; Feldhacker and Molitor, 2019).

Despite the assessment of the ^*^A Test and the RT in various populations, including in athletes of various sports, only one other study to date has measured the performance of athletes of these tasks under different conditions. A study by Bigsby et al. (2014) utilized a similar methodology in which they tested performance in these two tests on a Bosu Ball after assessing them on the ground in a team of collegiate American football players. The findings of the present study opposed the findings of the Bigsby et al. (2014) study, in which they found the football players performed worse during the dual-task condition atop the Bosu Ball. They also found this effect during the RT, whereas the present study found no differences in performance across the two conditions. Differences in these findings can likely be attributed to sport-specific demands; American football players act quickly with primarily planned actions, such as a lineman at the start of the snap, whereas the constant mobility of soccer players restricts them from cognitively processing during a game without the concurrent activation of their lower body.

Participants implemented different search strategies in different situations. Most notably, stances changed from more erect and casual postures during the ground tasks (*Endurance task, Single-load proactive, Single-load RT*) to a ready to react position of improved postural stability, exhibiting a lowered center of gravity in the dual load conditions. Anticipatory postural adjustments to stabilize the body when already in the ready to react position may facilitate quicker reaction times, particularly when in combination with a compliant surface (Dietz et al., 2000). This is similar to dual-task situations that arise on the playing field and is analogous to previous findings in which exercise was found to elicit an improvement in mean reaction time (Davranche et al., 2006; Pesce et al., 2007).

The tendency for the defender position to have among the lowest average choice reaction times can likely be attributed to the reactionary nature of the position; precognition and split-second decision-making are invaluable abilities for a player who must intercept, tackle, or otherwise disrupt attacking players. Similarly, players in the forward and midfielder positions must be ever conscious of shifting defenses, player mismatches, ball position, and be ready to capitalize from a limited opportunity. Goalies in the present study had a tendency for the slowest reaction times during proactive tasks; while the assessments of positional differences in traditional “choice reaction” assessments have primarily focused on goalies vs. field players, much research exists surrounding reactive agility in this population, of which goalies are typically much slower (Zemkova, 2016).

Albeit performance in choice reaction tasks, it is worth noting that, while failing to reach significance, goalies outperformed the other positions during the simple reaction tasks; this finding for simple reactivity performance is consistent with previous findings that support superior performance by goalkeepers (Ruschel et al., 2011; Taskin et al., 2016). Visual components of this task were comparable to the other positions, but motor performance tended to be among the fastest in both conditions, which contributed to the summative physical times for both components. This finding may reflect the positional differences in reliance on predictive cues; while it is undoubtedly vital for goalies to have strong choice reactivity, there is an increased likelihood for them to use situational cues to augment their decisions on the field as compared to the other positions. Goalies are able to recognize opponent and teammate arrangement, field positioning, and cue into the body movements of the opponents to predict the general region in which they will need to react. In this case, these predictive cues, if acknowledged quick enough, make this sport-specific action for goalies more similar to a simple reaction task, whereas the other positions should rely more heavily on choice reactivity. This warrants the need for further exploration of this concept with larger samples.

Another notable difference found in the goalkeepers was the differing longitudinal trends during the *Endurance* condition of proactive tasks. While goalkeepers responded with the fewest number of responses during each of the 4 min of the task, the longitudinal trend in performance failed to match that of the other positions toward the end of the task. As the defenders, midfielders, and forwards maintained or improved upon their number of responses with each additional minute, the goalkeepers dropped off in their responses after the second minute. When considering the differences in attentional requirements during gameplay, this is likely a result of the intermittent bursts of intense reactionary attention required by the goalkeeper position occurring only when the ball is nearing their goal. This situational requirement for maintaining reactivity for goalies is contrasted by the field positions, which continuously follow the ball, requiring sustained attentional span and reactivity for longer durations.

Despite the small sample size, results demonstrate considerable trends that provide insight into the performance of this population. While all positional groups were determined to have a normal distribution, having a sample of only three goalkeepers as compared to seven players of the other three positions could contribute to the divergence of findings to those of previous studies. Obtaining a larger sample of Division I collegiate Women's Soccer players is needed for further verification of the trends found herein of this preliminary evaluation of position-specific samples. It should be noted, however, that combining players from highly ranked teams to players from less qualified teams may further affect trends in the data.

While the present study did not obtain formal measures of head or eye movements, these along with additional measures of balance and the height of the center of mass could be beneficial in quantifying the changes in strategy and performance with an added physical load. For specific populations, namely athletes, visual training may aid in improving reaction time, which has been studied in a few small samples (Klavora et al., 1995; Feldhacker and Molitor, 2019; Feldhacker et al., 2019). Though, repeated training that requires concurrent cognitive loading and motor coordination may aid in bridging the gap for translatability between training and competition.

## Conclusion

Given the unique demands of soccer, frequent training of choice reactivity during physical activity and reactive agility is likely to contribute to sport-specific differences of athletes in other published literature. This probable effect of training adaptations improves the ability of soccer players to complete choice reactivity tasks as compared with other collegiate athletes. Further, the notable difference in positional demands between goalies and field players is an important consideration in the assessment of reactivity. While simple reactive performance in the present study mirrored previous findings in that no significant effects existed between positions, those of choice reactivity demonstrated a relatively novel discovery. Coupled with the limited research for positional differences, the lack of consistency for task complexity in assessments of choice reactivity makes it difficult to assess the effect size on task performance. However, in the present study, it is suggested that the poor performance demonstrated by goalies only in tasks of choice reactivity may be indicative of the translatability of the task to gameplay. The potential for predictive cues to factor into choice reactivity performance yields the need for further assessment surrounding reactivity in an athletic population.

## Data Availability Statement

The raw data supporting the conclusions of this article will be made available by the authors, without undue reservation.

## Ethics Statement

The studies involving human participants were reviewed and approved by West Virginia University Institutional Review Board. The patients/participants provided their written informed consent to participate in this study.

## Author Contributions

LR participated in study design and coordination, carried out participant consent and data collection, performed the statistical analysis, and drafted the manuscript. CB participated in study design and coordination, helped with data collection, and helped draft the manuscript. BR helped with statistical analysis and helped draft the manuscript. SG conceived of the study, participated in its design and coordination, and helped to draft the manuscript. All authors have read and approved the final version of the manuscript, agree with the order of presentation of the authors, and agree to be accountable for the content of the study.

## Conflict of Interest

The authors declare that the research was conducted in the absence of any commercial or financial relationships that could be construed as a potential conflict of interest.

## Publisher's Note

All claims expressed in this article are solely those of the authors and do not necessarily represent those of their affiliated organizations, or those of the publisher, the editors and the reviewers. Any product that may be evaluated in this article, or claim that may be made by its manufacturer, is not guaranteed or endorsed by the publisher.
